# RETRACTION: Effect of Ultrasound‐Supported Wound Debridement in Subjects with Diabetic Foot Ulcers: A Meta‐Analysis

**DOI:** 10.1111/iwj.70230

**Published:** 2025-02-11

## Abstract

**RETRACTION**: ChenH. , XiaoT. , ZhangL. , LiuN. , LiangX. , LiT. , WangJ. , PengY. , LiuY. , and XuJ. , “Effect of Ultrasound‐Supported Wound Debridement in Subjects with Diabetic Foot Ulcers: A Meta‐Analysis,” International Wound Journal 20, no. 7 (2023): 2618–2625, 10.1111/iwj.14133.36905211 PMC10410332

The above article, published online on 11 March 2023, in Wiley Online Library (http://onlinelibrary.wiley.com/), has been retracted by agreement between the journal Editor in Chief, Professor Keith Harding; and John Wiley & Sons Ltd. Following an investigation by the publisher, all parties have concluded that this article was accepted solely on the basis of a compromised peer review process. The editors have therefore decided to retract the article. The authors did not respond to our notice regarding the retraction.

**RETRACTION**: H. Chen , T. Xiao , L. Zhang , N. Liu , X. Liang , T. Li , J. Wang , Y. Peng , Y. Liu , and J. Xu , “Effect of Ultrasound‐Supported Wound Debridement in Subjects with Diabetic Foot Ulcers: A Meta‐Analysis,” International Wound Journal 20, no. 7 (2023): 2618–2625, 10.1111/iwj.14133.36905211 PMC10410332

The above article, published online on 11 March 2023, in Wiley Online Library (http://onlinelibrary.wiley.com/), has been retracted by agreement between the journal Editor in Chief, Professor Keith Harding; and John Wiley & Sons Ltd. Following an investigation by the publisher, all parties have concluded that this article was accepted solely on the basis of a compromised peer review process. The editors have therefore decided to retract the article. The authors did not respond to our notice regarding the retraction.

